# Evaluating geographic imputation approaches for zip code level data: an application to a study of pediatric diabetes

**DOI:** 10.1186/1476-072X-8-54

**Published:** 2009-10-08

**Authors:** James D Hibbert, Angela D Liese, Andrew Lawson, Dwayne E Porter, Robin C Puett, Debra Standiford, Lenna Liu, Dana Dabelea

**Affiliations:** 1 Department of Epidemiology and Biostatistics and Center for Research in Nutrition and Health Disparities, Arnold School of Public Health, University of South Carolina, 921 Assembly Street, Columbia, SC, USA; 2 Medical University of South Carolina College of Medicine, 135 Cannon Street, Suite 303, Charleston, SC, USA; 3 Department of Environmental Health Sciences, Arnold School of Public Health, University of South Carolina, 921 Assembly Street, Columbia, SC, USA; 4 Department of Epidemiology and Biostatistics, Arnold School of Public Health, University of South Carolina, 800 Sumter Street, Columbia, SC, USA; 5 South Carolina Cancer Prevention and Control Program, University of South Carolina, 915 Greene Street, Columbia, SC, USA; 6 Children's Hospital Medical Center, 3333 Burnet Avenue, Cincinnati, OH, USA; 7 University of Washington Child Health Institute, Seattle, WA, USA; 8 University of Colorado School of Public Health, 13001 East 17th Avenue, Denver, CO, USA

## Abstract

**Background:**

There is increasing interest in the study of place effects on health, facilitated in part by geographic information systems. Incomplete or missing address information reduces geocoding success. Several geographic imputation methods have been suggested to overcome this limitation. Accuracy evaluation of these methods can be focused at the level of individuals and at higher group-levels (e.g., spatial distribution).

**Methods:**

We evaluated the accuracy of eight geo-imputation methods for address allocation from ZIP codes to census tracts at the individual and group level. The spatial apportioning approaches underlying the imputation methods included four fixed (deterministic) and four random (stochastic) allocation methods using land area, total population, population under age 20, and race/ethnicity as weighting factors. Data included more than 2,000 geocoded cases of diabetes mellitus among youth aged 0-19 in four U.S. regions. The imputed distribution of cases across tracts was compared to the true distribution using a chi-squared statistic.

**Results:**

At the individual level, population-weighted (total or under age 20) fixed allocation showed the greatest level of accuracy, with correct census tract assignments averaging 30.01% across all regions, followed by the race/ethnicity-weighted random method (23.83%). The true distribution of cases across census tracts was that 58.2% of tracts exhibited no cases, 26.2% had one case, 9.5% had two cases, and less than 3% had three or more. This distribution was best captured by random allocation methods, with no significant differences (p-value > 0.90). However, significant differences in distributions based on fixed allocation methods were found (p-value < 0.0003).

**Conclusion:**

Fixed imputation methods seemed to yield greatest accuracy at the individual level, suggesting use for studies on area-level environmental exposures. Fixed methods result in artificial clusters in single census tracts. For studies focusing on spatial distribution of disease, random methods seemed superior, as they most closely replicated the true spatial distribution. When selecting an imputation approach, researchers should consider carefully the study aims.

## Background

There has long been recognition that place or geographic area can impact health behaviors and health outcomes [1-4]. The advent of geographic information system (GIS) technology and its widespread dissemination has enormously simplified the identification and characterization of place via address match geocoding, i.e. the assignment of geographic coordinates to a street address through interpolation based on a proportional distance between addresses in a record and an address range for a street segment [5].

The validity of epidemiological studies involving geocoded data relies on the proportion of cases that can be geocoded and on the positional accuracy of the geocodes [6]. Successful address match geocoding relies, in part, on the availability of complete and correct address information [2]. However, address information in combination with health attributes is often considered protected health information under the Health Insurance Portability and Accountability Act (HIPAA). Thus only limited address information, such as a ZIP code, may be available for research [7].

In the presence of missing or incomplete address data, investigators must decide whether to discard the incomplete data or, based on a variety of assumptions, allocate them to a representative location, e.g. a geometric center or centroid of the smallest geographic unit available, typically in the US, a ZIP code [8]. Discarding incomplete data ensures a database with a high level of accuracy, however may result in a significant reduction in total cases available for analysis. Furthermore, if incompleteness of address data is associated with other attributes under study (i.e. if incomplete data are spatially correlated or predominantly located in rural areas) exclusion could lead to a geographic selection bias [8].

Allocating cases to the smallest geographic unit available for all data points ensures that the database retains the maximum possible number of cases, although this method contains several drawbacks. When allocated to the centroid of a geographic unit, cases may fall into uninhabited areas such as lakes or national parks. Also, the geographic units themselves may vary greatly in size and location over a short period of time, as has been shown for postal ZIP codes in the United States (U.S.) [9].

Geo-imputation introduces a third option by using available address data in conjunction with assumptions based on available demographic or geographic data. Spatial apportionment of data has a long history of utilization in social sciences [10-14]. More recently, geo-imputation has become popular in epidemiological studies for allocation of individual study participants to geographic units [15,16]. Very little is known, however, with respect to the accuracy of geographic imputation methods [17].

The purpose of the current study was to evaluate the accuracy and utility of a variety of geo-imputation approaches for ZIP code data at the individual level (i.e. correct allocation of individual case to census tract) and at the group level (i.e. appropriate spatial distribution of cases across tracts). In the context of a project on the spatial epidemiology of diabetes, we used data from the SEARCH for Diabetes in Youth study [18]. We also aimed to describe the data at hand with respect to address completeness and geocoding success.

## Research methods

### Study Design

The present study was approved by Institutional Review Boards (IRB) from all participating entities and conducted using HIPAA compliant procedures. The SEARCH for Diabetes in Youth Study was initiated in 2000 to estimate the population prevalence, and incidence of all types of diabetes in youth in the U.S. by age, gender, race/ethnicity, and diabetes type in four geographically defined populations and two membership/health-plan-based populations using consistent methodology for case ascertainment and classification [18]. For the present study, data from the four geographic defined populations were included, which represent four distinct geographic U.S. regions of varying urban and rural characteristics, population densities, and socioeconomic status. Study sites included Colorado (all 64 counties), Ohio (six counties surrounding Cincinnati, OH, including two in Kentucky and one in Indiana), South Carolina (all 46 counties), and Washington (five counties surrounding Seattle, WA). The study areas varied widely with respect to urban and rural landscapes. Washington and Ohio were exclusively confined to the Seattle, WA and Cincinnati, OH areas respectively, which contained the highest mean population densities at the Census tract level per square kilometer (1379.22 and 1327.66 respectively). South Carolina contained the largest amount of rural landscape with a mean tract population density of 416.77 per square kilometer. The regional land area sizes varied from the 6,826 km^2 ^in the Ohio site to 269,736 km^2 ^in the Colorado site. Land area was calculated in ArcGIS 9.3 [19] using an equal area projection.

### Geocoding of data

The study population included 2,538 youth aged 0-19 years: 2,068 cases were diagnosed between 2002 and 2003 with type 1 and type 2 diabetes and 470 other diabetes cases that were part of a SEARCH case control study. Cases were geocoded based on street address (address matching), ZIP code, or county depending on the availability of address information. The 2000 TIGER (Topographically Integrated Geographic Encoding and Referencing) road network [20] was used for geocoding in ArcGIS 9.3 [19] and was complemented with Zip Code Tabulation Areas (ZCTA). The ZCTA was first used in the 2000 Census, and was created to overcome the difficulties in defining the land area encompassed by a ZIP code [20]. ZCTAs are created through the aggregation of Census blocks into areas that most closely correspond with ZIP code areas [9].

Due to Internal Review Board (IRB) logistics, nearly 42% of the cases in the Washington site were restricted to ZIP code only (Table 1). A significant number of full addresses available in South Carolina could not be geocoded to the street address level, as these could not be located using 2000 TIGER. 2006 TIGER incorporated more recent changes in the road network, improving the geocoding effort. Thus, geocoding for South Carolina was completed using a combination of TIGER years. The 2000 TIGER centerlines were selected for geocoding in order to more closely match the years in which the case data was collected as street names and ZIP codes may change frequently over time [9].

**Table 1:** Data completeness and geocoding success by site

	**Colorado**	**Ohio**	**South Carolina**	**Washington**
	
**Total Cases**	**1003**	**360**	**666**	**509**
	***n (%)***	***n (%)***	***n (%)***	***n (%)***
Full Address Available	943 (94.0%)	333 (92.5%)	512 (76.9%)	295 (58.0%)
POBOX/RR Address	27 (2.7%)	2 (0.5%)	42 (6.4%)	5 (1.0%)
Missing Address (ZIP code only)	33 (3.3%)	25 (7%)	110 (16.7%)	209 (41.0%)
Geocoded Full Address	867 (86.4%)	322 (89.5%)	452 (67.9%)	290 (57.0%)

### Data Cleaning and Quality

In a first step, topological anomalies in the ZCTA boundaries were removed. While the ZCTA files contain polygons for individual ZIP codes, water bodies and areas where no addressable postal locations existed were also contained in the file. Unlike other statistical entities from the Census, such as a tract or block group, ZCTAs do not necessarily require a contiguous boundary. This means that a given ZCTA may actually be composed of two or more noncontiguous polygons [20]. These anomalies in the ZCTA boundaries file were dealt with using an approach similar to that taken by Grubesic and Matisziw [21], whereby polygons identified by the Census as water polygons and polygons containing no addresses were removed. ZCTAs composed of multiple polygons were dissolved into a single polygon based on a common ZIP code.

### Calculation of Census Tract Weighting Factors

**Figure 1:**
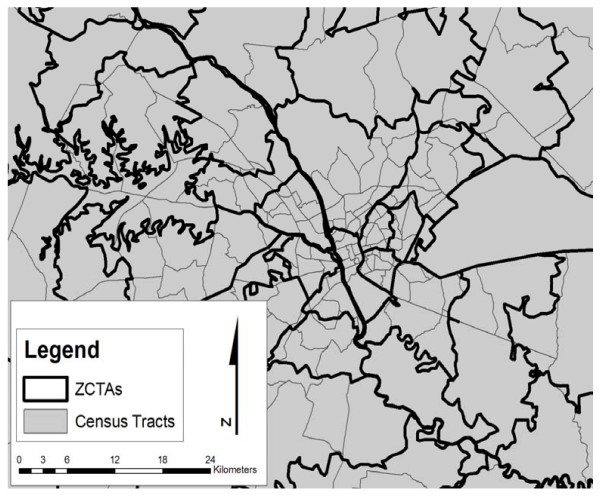
**ZCTA and Census tract boundaries**.

As shown in Figure 1, ZCTAs do not conform to census tract boundaries and generally cover a larger spatial area than a census tract. The proportion of overlap between the ZCTAs and the census tracts was utilized to obtain either land area-based or population-based weighting factors that were subsequently used in geo-imputation. Each ZCTA was subdivided by the tracts overlapped using geoprocessing components within a GIS. The geometric intersections of ZCTAs and tracts were computed and the tracts (or portions thereof) were joined with the attributes of the ZCTAs.

For the calculation of land-area weighting factors, the land area of a given tract that overlapped with a ZCTA was obtained from geoprocessing output and expressed as the proportion of the total ZCTA area. Table 2 illustrates this approach for ZCTA 29001 which contained five individual tracts. The weight is determined by dividing the land area of each tract within the ZCTA by the total ZCTA land area.

**Table 2:** Weighting by land area

**ZCTA**	**ZCTA Area (km^**2**^)**	**Tract ID**	**Tract Area in ZCTA (km^**2**^)**	**Proportion Tract Area in ZCTA**
29001	202.54	T_1_	171.37	0.84
29001	202.54	T_2_	15.75	0.07
29001	202.54	T_3_	9.77	0.05
29001	202.54	T_4_	5.61	0.03
29001	202.54	T_5_	0.03	0.01

**Figure 2:**
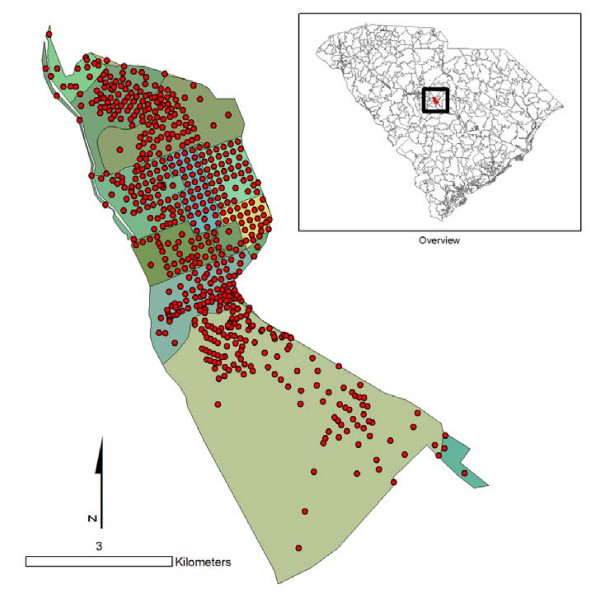
**Block centroids and tracts within a ZCTA**.

**Figure 3:**
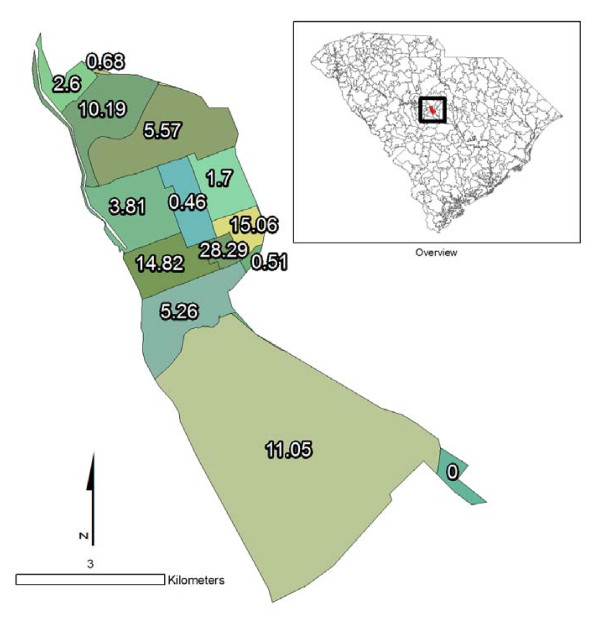
**Weighting of tracts within a ZCTA**.

For the calculation of population-based weights, data were used from the block level Census Summary File 1 (SF1) [22]. First, the total population was calculated for each ZCTA by summing the population estimates for all census blocks contained within a ZCTA. Blocks are contiguous with ZCTA boundaries (Figure 2) and were used to calculate census demographic data for each ZCTA and each tract proportion (Figure 3). Two types of population-based weights were investigated, based either on total population or on population 19 years or below. The population aged 0-19 was calculated using a summation of SF1 variables: male under 5 years (P012003), male 5 to 9 years (P012004), male 10 to 14 years (P12005), male 15 to 17 years (P012006), male 18 and 19 years (P012007), female under 5 years (P012027), female 5 to 9 years (P012028), female 10 to 14 years (P012029), female 15 to 17 years (P012030), and female 18 and 19 years (P012031). Total population was imported from variable P001001. Tract proportions containing zero population received a weight of zero (Figure 3) and were not considered in any population-weighted imputation.

### Geo-imputation methods

Two general types of geo-imputation methods were evaluated including fixed (deterministic) and random (stochastic) geo-imputation approaches. For each of these, both population and area based weighting factors were applied.

For the fixed allocation approaches, all cases within a ZCTA were allocated to the tract with the largest weighting factor as described above (i.e. area, total population, or total youth population weighting factor). These methods are abbreviated in the text and tables as a) *FixedArea*: Fixed area-weighted allocation; b) *FixedPop*: Fixed total population-weighted allocation; and c) *Fixed019*: Fixed population-weighted using 0-19 age group. In addition, we performed the most commonly used fixed allocation method which allocates a case to the ZIP centroid, which was designated d) *FixedZip*.

The random allocation approaches used methods similar to those described by Henry and Boscoe [17]. The weights obtained either from land-area or population-based calculations described above represented the chance of being allocated to a tract [17]. For *n *tracts within a ZCTA, there will be *n *proportions. Let us assume that there are five tracts (*T*_*1...5*_) overlapping ZCTA *Z*_1 _and that their proportional contributions (for either area or population) are 0.84, 0.7, 0.5, 0.3 and 0.1 respectively. For each tract, a range is created using proportion weights (Table 3). Subsequently, a random number [0.0-1.0] is generated. Each case is then allocated to the tract that contains the range of weights into which the random number is contained. This approach results in the probability of an assignment in a particular tract being equivalent to the proportion of the metric being evaluated, e.g., area-weighted. For example, the range of T_1 _(Table 3) of 0.00-0.84 results in an 84% chance of a case randomly assigned to T_1_

**Table 3:** Weighting and ranges for allocation to tracts

**Tract ID**	**Tract Area in ZCTA (km^**2**^)**	**Proportion Tract Area in ZCTA**	**Cumulative Proportion**	**Range**
T_1_	171.37	0.84	0.84	0.00 - 0.84
T_2_	15.75	0.07	0.91	0.84 - 0.91
T_3_	9.77	0.05	0.96	0.91 - 0.96
T_4_	5.61	0.03	0.99	0.96 - 0.99
T_5_	0.03	0.01	1.00	0.99 - 1.00

The random allocation methods are abbreviated in text and table as a) *RandArea*: Random area-weighted allocation; b) *RandPop*: Random total population-weighted allocation; c) *Rand019*: Random population-weighted using 0-19 year age group; and *RandRace019*: Random method using allocation by population distribution of 0-19 year old population by race/ethnicity. Race/ethnicity groups considered included non-Hispanic white, African American, Asian, Native American, and multi-ethnic/other. These categories represented all possible groups within the dataset.

### Statistical methods

Data are presented descriptively as percents and absolute numbers. Individual level accuracy assessments are represented as percent cases allocated correctly to a tract through geo-imputation methods. The distribution of cases to tracts achieved by the allocation methods was compared to the true distribution using the Chi-square statistic.

## Results

Address data characteristics and geocoding characteristics are summarized in Table 1. No site had complete address information for all cases, but both Colorado and Ohio had a markedly higher proportion of full addresses available than South Carolina and Washington, which were unable to obtain full addresses on a fraction of cases due to HIPAA related restrictions. An address is considered to be full if it contains a street number, street name, street type and ZIP code. South Carolina had a markedly higher number of addresses with PO Box or RR (rural route) designations. Both the Ohio and Colorado sites had the overall highest proportion of successfully geocoded addresses (CO = 86.4%, OH = 89.5%) The geocoding success rate (expressed as a proportion of full addresses available) was highly consistent across sites ranging from 92% in Colorado, 97% Ohio, 88% in South Carolina, and 98% in Washington.

To evaluate the various geo-imputation methods, the dataset was limited to those cases with a geocoded full address (total 1,931 cases). Each of the eight allocation methods were applied to the site-specific data assuming that the only available piece of address information available was a ZIP code (i.e. a worst case scenario) and then compared with the known, true location.

Table 4 summarizes the individual-level accuracy of the imputation approaches. The *Fixed019 *and *FixedPop *methods performed best at the individual level, with identical results in all sites except South Carolina. The proportion of cases correctly assigned to their census tract ranged from 23% to 37% across the sites (overall mean 30.26%). The commonly used *FixedZIP *method, the *FixedArea *method and the *RandArea *method performed extremely poorly. The *RandRace019 *method saw a slight improvement when compared to the other random allocation methods at the individual level for three of the four sites. However we observed a 5% reduction in accuracy in the Washington site with *RandRace019*.

**Table 4:** Individual level accuracy of fixed and random geo-imputation methods by site

	**Geo-imputation methods**							
	
	**FixedZip**	**FixedArea**	**FixedPop**	**Fixed019**	**RandArea**	**RandPop**	**Rand019**	**RandRace019**
	***%***	***%***	***%***	***%***	***%***	***%***	***%***	***%***
Colorado	16.77	14.44	23.03	23.03	13.94	21.11	19.80	21.40
Ohio	21.12	22.98	33.54	33.54	21.43	20.50	21.12	25.50
South Carolina	26.72	30.34	37.21	35.69	25.57	28.63	27.29	30.13
Washington	21.72	16.21	27.24	27.24	14.83	20.34	22.76	18.30

Results of the evaluation of group level accuracy are summarized in Additional File 1. The column entitled "*True*" lists the number of tracts that contain a given number of cases ranging from 0 to greater than 5. Given that diabetes in youth is a rare condition and our study was focused on incident cases, it was not surprising that across the entire study area more than 50% of all tracts did not contain a single case. In general, between 24 and 29% of tracts contained a single case with a sequentially decreasing proportion of tracts containing multiple cases. The remainder of the table describes the allocation of cases to tracts achieved by each of the eight imputation methods.

The distribution of cases across tracts was then compared using the Chi-square statistic (Table 5). Significant differences were observed between the distribution achieved by the *FixedZip*, *FixedArea*, *FixedPop *and *Fixed019 *imputation methods compared to the true distributions observed in our data. In contrast, none of the four random allocation methods seemed to differ significantly from true allocation, which suggests that these methods are superior to any of the fixed methods at the level of group accuracy. Both the *Rand019 *and *RandPop *methods performed similarly, with the youth population weighting being somewhat advantageous in South Carolina, Ohio and Washington.

**Table 5:** Chi-square statistics associated with group level accuracy

	**Geo-imputation methods**							
	
	**FixedZip**	**FixedArea**	**FixedPop**	**Fixed019**	**RandArea**	**RandPop**	**Rand019**	**RandRace019**
								
Colorado	399.4479	427.7909	388.6003	386.8368	7.5191	1.038	1.2907	3.7910
	p < 0.0001	p < 0.0001	p < 0.0001	p < 0.0001	p = 0.1848	p = 0.9594	p = 0.9359	p = 0.5799
								
Ohio	141.5495	152.4194	139.2934	139.2934	3.0906	1.362	1.2907	1.8665
	p < 0.0001	p < 0.0001	p < 0.0001	p < 0.0001	p = 0.686	p = 0.9594	p = 0.9359	p = 0.8673
								
South Carolina	146.8333	141.8189	149.1956	143.6908	4.3042	7.6184	1.7513	1.0542
	p < 0.0001	p < 0.0001	p < 0.0001	p < 0.0001	p = 0.5065	p = 0.1786	p = 0.8824	p = 0.9580
								
Washington	146.5466	23.6656	22.777	129.8429	1.4884	1.1255	0.2134	3.8452
	p < 0.0001	p = 0.0003	p = 0.0004	p < 0.0001	p = 0.9144	p = 0.9518	p = 0.999	p = 0.5719

## Discussion

The individual level accuracy of eight imputation methods was assessed for over 2,000 cases of diabetes across four U.S. regions. This study is among the few to determine accuracy of geo-imputation methods using collected clinical data that had been geocoded through HIPAA compliant procedures. The vast majority of published epidemiologic work to date that has dealt with incomplete address information has reported allocating missing data to ZIP code centroid [9,23,24]. This can be problematic as ZIP codes are less spatiotemporally stable than Census statistical areas such as tracts or block groups [9]. Investigators should pay particular attention when comparing identical ZIP codes from datasets that are temporally dissimilar.

At the level of individual assignment, fixed population-weighted methods showed a mean accuracy of 30.26% (Min 23.03%, Max 33.54% using total population weight) and 30.45% (Min 23.03%, Max 37.98% using youth population ages 0-19 weight). Although these geo-imputation methods led to a disproportionate number of cases allocated to a single tract within a ZCTA, instances exist where this method would be useful. Heavily urbanized residential areas with high population density will contain tracts and ZCTAs smaller in land area and simplify distance calculation to exposure sites [25].

Although the individual case accuracy of the random methods was lower than fixed methods, randomization allowed for each tract in a ZCTA to have a chance of a case being allocated to it. This allowed for a distribution more closely approximating that seen in reality (i.e. the *True *column in Additional File 1). Randomized allocation applied to the youth population from Census SF1 was found to provide the best approximation of the true distribution of cases within census tracts for all sites.

Individual accuracy of all methods varied geographically. Colorado results were lowest among most of the eight methods. Colorado comprised the largest total land area and South Carolina was the least densely populated of the four sites. Tract size for Colorado was also largest, averaging 254 km^2^. Interestingly, it was anticipated that sites containing tracts of smaller land area achieve highest accuracy with Washington and Ohio being smallest with average tract areas of 29.53 km^2 ^and 26.14 km^2^ respectively. However, South Carolina (average tract area 92.32 km^2^) results were consistently highest among all eight methods with Ohio and Washington being 2^nd ^or 3^rd ^when comparing each method's accuracy across sites (Table 4).

Compared to the fixed allocation methods, random population-weighted methods showed a mean accuracy of 22.64% at the individual level (Min 20.34%, Max 28.63% using total population weights), 21.07% (Min 17.47%, Max 26.72% using youth population ages 0-19 weights) and 23.83% (Min 18.30, Max 30.13) using youth population and race/ethnicity. Henry and Boscoe [17] saw a similar accuracy of 25.9% using total population as a weighting mechanism.

At the level of group accuracy, the *RandPop *and *Rand019 *methods performed similarly across all sites except Colorado, with *RandPop *(p = 0.9594) being slightly better than *Rand019 *(p = 0.9359) and South Carolina with the *RandRace019 *performing best (p = 0.9580). This may be due in part to both the rural nature of South Carolina, and to the larger amount of people over 65, particularly within coastal areas. *RandArea *performed the poorest across all sites when compared to the true distribution. To the best of our knowledge, this is the first paper to evaluate the ability of geo-imputation approaches to approximate distribution of cases across space.

In our study geography, a ZIP code overlapped with a median number of 4 (minimum 1, maximum 29) Census tracts. This relationship in fact sets a sort of upper limit on the individual-level accuracy of any imputation method, because as the number of tracts per ZIP code increases, the likelihood of correct assignment of an individual decreases, hence, the low overall magnitude of the individual level accuracy of the geo-imputation methods. Furthermore, this relationship between ZIP codes and tracts is likely responsible for the fact that in our data, the fixed allocation methods performed better than any of the random allocation methods at the individual level.

Henry and Boscoe [17] showed that weighting using multiple covariates such as race/ethnicity in addition to age achieves higher accuracy. Correspondingly, we refined the weighting using the population of youth aged 0-19 years by additionally considering the race/ethnic composition of the population of youth. Consistent with previous findings, this approach produced a slight increase in accuracy in the Colorado, Ohio, and South Carolina study sites at the individual level. However, the Washington site experienced a 4% drop in accuracy when accounting for race. It is conceivable that in the Washington site, both the lower levels of residential racial segregation in urban Seattle plus the larger ethnic and multi-racial diversity of the Seattle population contribute to the loss in specificity of an assignment, thereby increasing inaccuracy.

It is important to note that the geo-imputation methods shown were conducted entirely within the GIS framework and utilized custom tools developed to handle the random allocation and extend the capabilities of the GIS. Although it is entirely possible to use purely statistical allocation, GIS was essential to both the rapid implementation of the geo-imputation methods as well as the weighting calculations, particularly the area-based weights. Investigators wishing to use geo-imputation methods should take into account the benefits offered in these software packages. Investigators may contact the author to obtain the tool created to perform the geo-imputations presented in this paper.

It has been well established that geocoding success rate can differ significantly with respect to urban and rural areas and can be seen as being correlated with population density [6,25,26]. Since address match geocoding is accomplished through interpolation along a street segment, a longer segment common to rural areas may introduce greater error. Furthermore, addresses drawn from rural areas are more likely to contain PO Boxes or Rural Routes as address information, confounding the geocoding process [27].

A fundamental, very conservative assumption of the present analysis is that a ZIP code is the only address portion available on the entire data set. In many instances geoimputation would only be applied to the non-geocodable subset of the addresses. Addresses lacking other portions of a geocodable address (in this case, street number, street name, street type) would likely produce different results using these imputation methods. Furthermore, geo-imputation cannot fully compensate for low-quality address data, although it can provide a valuable solution in instances where an analysis will be conducted at spatial units smaller than those available for all cases. Other methods such as dasymetric mapping [28,29], manual intervention/interactive geocoding or re-coding using a different geocoding strategy may in some instances be preferable [30].

Although ZCTAs are used by the Census to represent the land area covered by a ZIP code, investigators must consider the potential for spatiotemporal mismatch of current ZIP codes to Census derived ZCTAs [9]. Since the primary function of ZIP codes is to aid the USPS in efficient mail delivery, it is necessary that ZIP codes be updated frequently between Census dates to reflect changes in population and the changes may not be well documented [7].

## Conclusion

In summary, our evaluation of geo-imputation approaches for ZIP code level data indicates that while fixed imputation methods yield the greatest accuracy at the individual level, random methods most closely replicate the true distribution of locations across space. Our study illustrates the wide range of geo-imputation approaches that may be considered above and beyond the commonly used ZIP code centroid method. It remains up to the investigator to fully understand the implications of handling missing address data with the methods available and to carefully consider the purpose of the study when selecting an imputation approach.

## Competing interests

The authors declare that they have no competing interests.

## Authors' contributions

JH acquired the data, analyzed and interpreted the data, drafted and revised the manuscript.

ALi contributed to acquiring the data and design of the study and drafted parts of the manuscript and made critical revisions to the manuscript. ALa made contributions to conception and design and revision of the manuscript. DP made contributions to conception and design and revision of the manuscript. RP contributed to the analysis and interpretation of the data and revisions of the manuscript. DS, LL, and DD contributed to acquiring the data and critical revision of the manuscript. All authors read and approved the final manuscript.

## Supplementary Material

Additional file 1 **Group level geo-imputation accuracy**. Table summarizing group level geo-imputation accuracy across all four study sites. Click here for file
