# Supplementary material for: Evaluating geographic imputation approaches for zip code level data: an application to a study of pediatric diabetes
Source: Int J Health Geogr. 2009 Oct 8;8:54. doi: 10.1186/1476-072X-8-54 (PMC2763852; doi:10.1186/1476-072X-8-54)
Supplement: Additional file 1 — Group level geo-imputation accuracy. Table summarizing group level geo-imputation accuracy across all four study sites. [file 1476-072X-8-54-S1.DOC]

|  | | Truth | | Geo-imputation methods | | | | | | | |  |
| --- | --- | --- | --- | --- | --- | --- | --- | --- | --- | --- | --- | --- |
|  | |  | | FixedZip | FixedArea | FixedPop | Fixed019 | RandArea | RandPop | Rand019 | RandRace019 | |
| Colorado | | | |  |  |  |  |  |  |  | |  |
|  | Cases† | | *n* (%)* | *n* (%)* | *n* (%)* | *n*(%)* | *n* (%)* | *n* (%)* | *n* (%)* | *n* (%)* | | *n*(%)* |
| 0 | | | 532 (50.09) | 889 (83.71) | 885 (83.33) | 885 (83.33) | 907 (85.40) | 572 (53.86) | 531 (50.00) | 541 (50.94) | | 539 (50.75) |
| 1 | | | 283 (26.65) | 55 (5.18) | 57 (5.37) | 58 (5.46) | 48 (4.52) | 261 (24.58) | 284 (26.74) | 286 (26.93) | | 283 (26.65) |
| 2 | | | 126 (11.86) | 11 (1.04) | 16 (1.51) | 16 (1.51) | 13 (1.22) | 124 (11.68) | 123 (11.58) | 120 (11.30) | | 140 (13.18) |
| 3 | | | 64 (6.03) | 20 (1.88) | 18 (1.70) | 18 (1.69) | 18 (1.69) | 47 (4.43) | 73 (6.87) | 54 (5.09) | | 61 (5.74) |
| 4 | | | 35 (3.30) | 14 (1.32) | 12 (1.13) | 12 (1.13) | 6 (0.56) | 27 (2.54) | 30 (2.82) | 36 (3.39) | | 23 (2.17) |
| 5+ | | | 22 (2.07) | 73 (6.87) | 74 (7.00) | 74 (7.00) | 70 (6.59) | 31 (2.92) | 21 (1.98) | 25 (2.35) | | 27 (2.54) |
| Ohio | | | |  |  |  |  |  |  |  | |  |
|  | Cases† | | *n* (%)* | *n* (%)* | *n* (%)* | *n*(%)* | *n* (%)* | *n* (%)* | *n* (%)* | *n* (%)* | | *n* (%)* |
| 0 | | | 249 (54.13) | 378 (82.17) | 374 (81.30) | 374 (81.30) | 413 (89.78) | 267 (58.04) | 262 (56.96) | 248 (53.91) | | 253 (55.00) |
| 1 | | | 135 (29.35) | 25 (5.43) | 25 (5.43) | 25 (5.43) | 25 (5.43) | 116 (25.22) | 121 (26.30) | 129 (28.04) | | 130 (28.26) |
| 2 | | | 53 (11.52) | 17 (3.70) | 18 (3.91) | 18 (3.91) | 18 (3.91) | 50 (10.87) | 54 (11.74) | 60 (13.04) | | 59 (12.83) |
| 3 | | | 15 (3.26) | 9 (1.96) | 10 (2.17) | 10 (2.17) | 10 (2.17) | 19 (4.13) | 16 (3.48) | 19 (4.13) | | 15 (3.26) |
| 4 | | | 5(1.09) | 9 (1.96) | 9 (1.96) | 9 (1.96) | 9( 1.96) | 7 (1.52) | 6 (1.30) | 4 (0.87) | | 2 (0.43) |
| 5+ | | | 3 (0.65) | 26 (5.65) | 27 (5.87) | 27 (5.87) | 26 (5.65) | 4 (0.87) | 4 (0.87) | 0 (0.00) | | 4 (0.87) |
| South Carolina | | | |  |  |  |  |  |  |  | |  |
|  | Cases† | | *n* (%)* | *n* (%)* | *n* (%)* | *n*(%)* | *n* (%)* | *n* (%)* | *n* (%)* | *n* (%)* | | *n* (%)* |
| 0 | | | 526 (60.67) | 690 (79.58) | 687 (79.24) | 685 (79.01) | 687 (79.24) | 549 (63.32) | 510 (58.82) | 513 (59.17) | | 523 (60.32) |
| 1 | | | 221 (25.49) | 57 (6.57) | 57 (6.57) | 58 (6.69) | 59 (6.81) | 191 (22.03) | 232 (26.76) | 236 (27.22) | | 228 (26.30) |
| 2 | | | 84 (9.69) | 53 (6.11) | 53 (6.11) | 54 (6.23) | 53 (6.11) | 81 (9.34) | 92 (10.61) | 83 (9.57) | | 78 (9.00) |
| 3 | | | 19 (2.19) | 15 (1.73) | 17 (1.96) | 17 (1.96) | 17 (1.96) | 27 (3.11) | 27 (3.11) | 22 (2.54) | | 23 (2.65) |
| 4 | | | 10 (1.15) | 17 (1.96) | 18 (2.08) | 18 (2.08) | 17(1.96) | 12 (1.38) | 4 (0.46) | 9 (1.04) | | 10 (1.15) |
| 5+ | | | 7 (0.81) | 35 (4.04) | 35 (4.04) | 35 (4.04) | 34 (3.92) | 7 (0.81) | 2 (0.23) | 4 (0.46) | | 5 (0.58) |
| Washington | | | |  |  |  |  |  |  |  | |  |
|  | Cases† | | *n* (%)* | *n* (%)* | *n* (%)* | *n*(%)* | *n* (%)* | *n* (%)* | *n* (%)* | *n* (%)* | | *n* (%)* |
| 0 | | | 518 (69.16) | 652 (87.05) | 585 (78.10) | 634 (84.65) | 594 (79.31) | 538 (71.83) | 523 (69.83) | 521 (69.56) | | 522 (68.41) |
| 1 | | | 184 (24.57) | 37 (4.94) | 161 (21.50) | 40 (5.34) | 147 (19.67) | 175 (23.36) | 197 (26.30) | 178 (23.77) | | 200 (26.21) |
| 2 | | | 37 (4.94) | 31 (4.14) | 10 (1.34) | 32 (4.27) | 13 (1.74) | 38 (5.07) | 36 (4.81) | 39 (5.21) | | 33 (4.33) |
| 3 | | | 9 (1.20) | 13 (1.74) | 5 (0.67) | 13 (1.74) | 5 (0.67) | 9 (1.20) | 7 (0.93) | 10 (1.34) | | 8 (1.05) |
| 4 | | | 1 (0.13) | 14 (1.87) | 2 (0.27) | 16 (2.14) | 4 (0.53) | 3 (0.40) | 0 (0.00) | 1 (0.13) | | 0 (0.00) |
| 5+ | | | 0 (0.00) | 24 (3.20) | 0 (0.00) | 14 (1.87) | 0 (0.00) | 0 (0.00) | 0 (0.00) | 0 (0.00) | | 0 (0.00) |
| † Cases per census tract  * Number of census tracts (percent) | | | | | | | | | | | |  |

Additional File 1. Group level geo-imputation accuracy.
